# A Case of a Durable Partial Response to Late‐Line Axitinib Following Prior Use of Tyrosine Kinase Inhibitors in Metastatic Papillary Renal Cell Carcinoma

**DOI:** 10.1002/iju5.70115

**Published:** 2025-11-14

**Authors:** Naoto Hodotsuka, Yasutomo Suzuki, Shohei Yamaki, Yuichiro Honda, Kyota Suzuki, Shuma Endo, Eigo Kuribayashi, Yukihiro Kondo

**Affiliations:** ^1^ Department of Urology Nippon Medical School Chiba Hokusoh Hospital Chiba Japan; ^2^ Department of Urology Nippon Medical School Hospital Tokyo Japan

**Keywords:** axitinib, computed tomography, renal cell carcinoma, tyrosine kinase inhibitor, vascular endothelial growth factor

## Abstract

**Introduction:**

Metastatic papillary renal cell carcinoma (PRCC) is associated with a poor prognosis. Many patients with PRCC respond to first‐ and second‐line systemic chemotherapy treatments; however, few respond to late‐sequence treatment. We describe a case of a long‐term response to fourth‐line axitinib after the use of tyrosine kinase inhibitor therapy for metastatic papillary renal cell carcinoma.

**Case Presentation:**

A 37‐year‐old male presented with hematuria and left back pain. Following systemic examination, he was diagnosed with left renal cell carcinoma cT3aN0M1. He underwent left radical nephrectomy. The pathological diagnosis was PRCC type 2. After using sunitinib, cabozantinib, and nivolumab, axitinib was selected as fourth‐line systemic therapy, resulting in a reduction in multiple liver metastases and 15 months of response.

**Conclusion:**

Late‐sequence treatment with axitinib following prior use of tyrosine kinase inhibitor therapy resulted in a long‐term response in a patient with metastatic papillary renal cell carcinoma.

**Abbreviations**
nccRCC: non‐clear cell renal cell carcinoma
OS: overall survival
PFS: progression‐free survival
PRCC: papillary renal cell carcinoma
WHO: World Health Organization

**Summary**

- Metastatic papillary renal carcinoma has a poor prognosis.
- Numerous patients respond to early‐sequence treatment; however, few respond to late‐sequence treatment.
- We report a case of a long‐term response to fourth‐line treatment with axitinib following the use of tyrosine kinase inhibitors.

## 1 Introduction

Papillary renal cell carcinoma (PRCC) is the most common subtype of non‐clear cell renal cell carcinoma (nccRCC) and is histologically classified into types 1 and 2. Type 2 has been associated with a worse prognosis than Type 1. Since the 2022 World Health Organization (WHO) classification, PRCC has been consolidated into a single type [1]. Currently, there is no established standard of care for systemic treatment.

Many patients with PRCC respond to first‐ and second‐line systemic chemotherapy treatments; however, few respond to late‐sequence treatment. Herein, we report a case of metastatic PRCC that showed a long‐term response (15 months) to fourth‐line axitinib after the use of sunitinib, cabozantinib, and nivolumab.

## 2 Case Presentation

**FIGURE 1:**
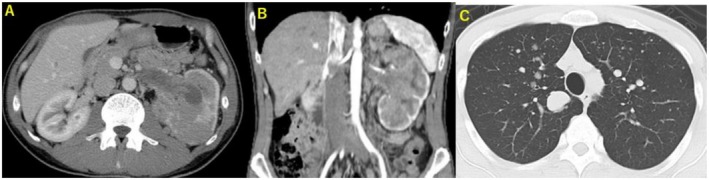
At initial presentation. (A, B) Contrast‐enhanced CT showed a poorly enhancing tumor extending into the left renal vein. (C) Chest CT showed multiple lung metastases. CT, computed tomography.

A 37‐year‐old male presented with gross hematuria and left flank pain. Contrast‐enhanced computed tomography showed a poorly enhancing left renal tumor and multiple lung metastases (Figure 1). The patient was diagnosed with left renal cell carcinoma cT3aN0M1 and underwent open left radical nephrectomy.

**FIGURE 2:**
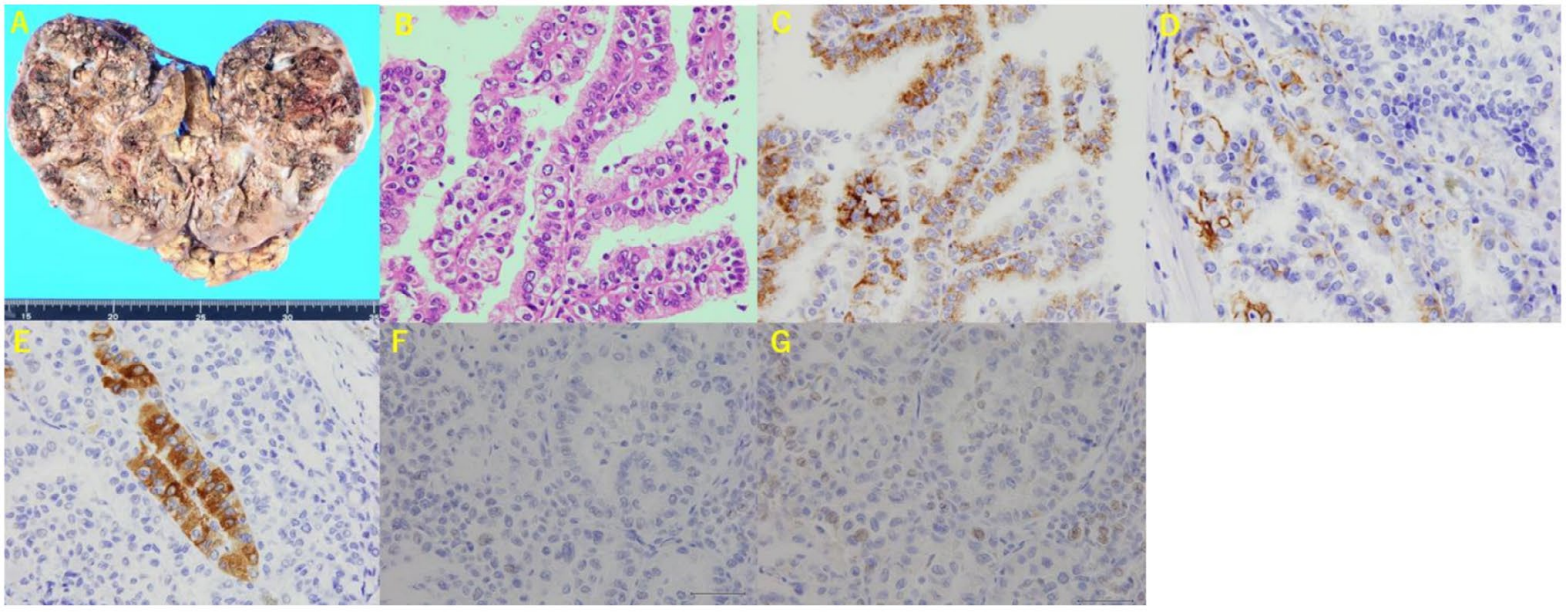
(A) Macroscopic findings (cut surface). Red arrows indicate the location of the renal vein embolism. (B) H&E staining. (C) Alpha‐methylacyl‐CoA racemase. (D) CKAE1/AE3. (E) MLANA. (F) TFE3. (G)TFEB. CKAE1/AE3, cytokeratin AE1/AE3; H&E, hematoxylin and eosin; MLANA, melan‐A; TFE3, transcription factor binding to IGHM enhancer 3; TFEB, transcription factor EB.

Macroscopically, the tumor presented with a multinodular appearance and showed hemorrhage and necrosis. Tumor thrombus was observed in the renal vein (Figure 2A). Histological examination revealed an abundance of tumor cells with eosinophilic‐to‐clear cytoplasm; some were small with scant cytoplasm, showing a predominantly papillary pattern with partial solid growth.

Immunohistochemical staining showed tumor cell positivity for p504S (alpha‐methylacy‐CoA racemase) and focal positivity for cytokeratin AE1/AE3 and melan‐A. Negativite result was observed for CK7, human melanoma black‐45 (HMB‐45), and Wilms tumor 1. Fumarate hydratase was not performed.

Given these findings, the patient's young age, and rapid progression, the initial pathological diagnosis at our hospital was MiT family translocation renal cell carcinoma, pT3a, pN1, and Grade 3. Due to the rarity of cancer and the lack of an established first‐line regimen, the patient was referred for a second opinion at a rare cancer center. Pathological reevaluation and comprehensive genomic profiling (CGP) were performed to explore potential clinical trials. Additional immunohistochemical staining was conducted for transcription factor binding to IGHM enhancer 3 and transcription factor EB, and both tests yielded negative results (Figure 2B–G). Based on these findings, the diagnosis was PRCC type 2, pT3a, pN1, and Grade 3. According to the International Metastatic RCC Database Consortium classification, the risk was intermediate based on one criterion: initiation of treatment within 1 year of diagnosis. CGP testing demonstrated a microsatellite stable status and tumor mutation burden of 0 mutations per megabase. CDKN2A, CDKN2B, and CDKN2C losses were detected. However, a suitable clinical trial was not found.

First‐line systemic treatment with sunitinib (50 mg/day) was initiated for 4 weeks, followed by a 2‐week interval. Lung metastases showed a partial response. At 5 months after treatment initiation, the patient developed a cerebellar hemorrhage. Head magnetic resonance imaging revealed multiple brain metastases. At 6 months, an increase in lung metastases was observed. Gamma knife therapy was performed for brain metastases.

**FIGURE 3:**
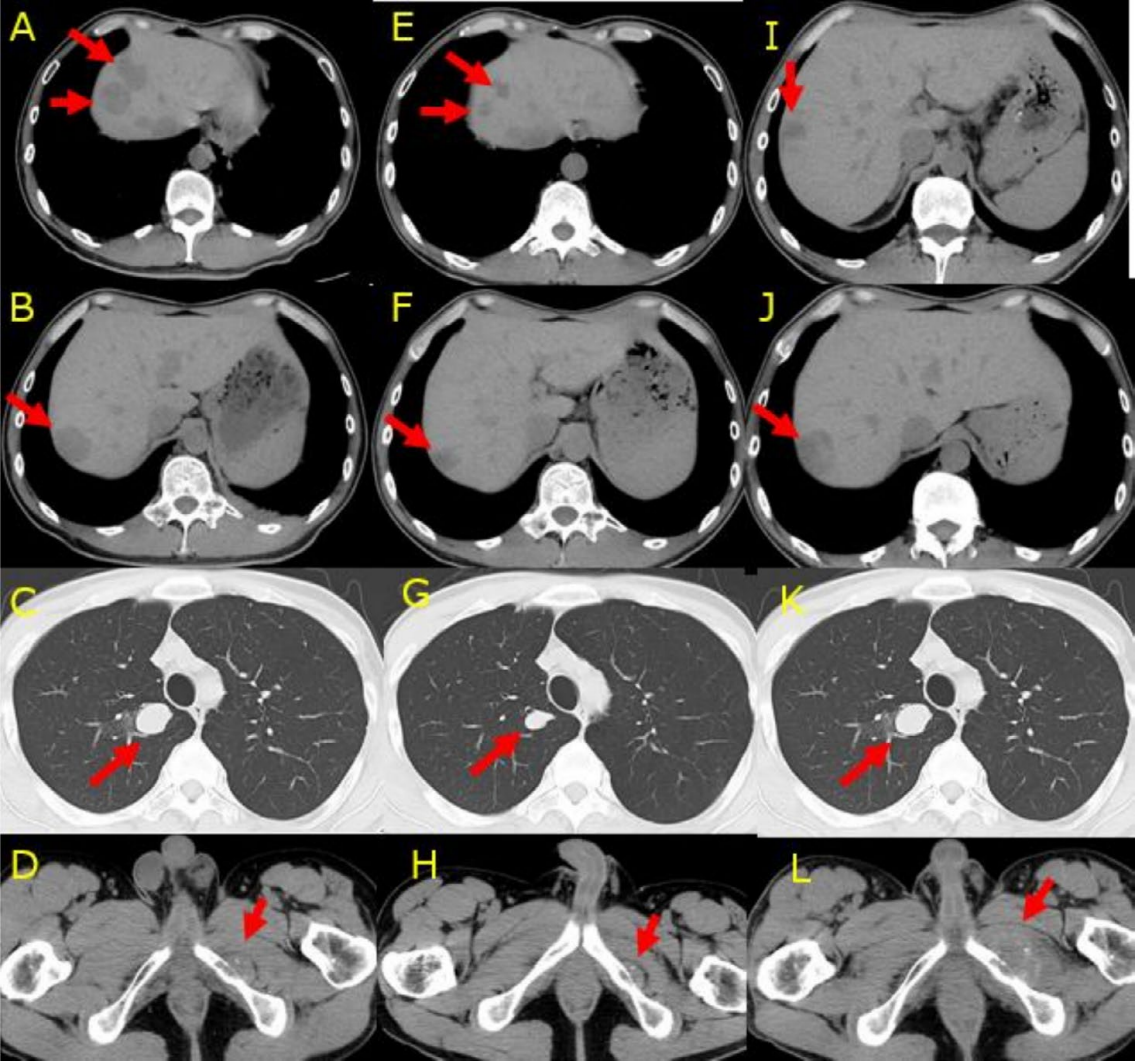
(A–D) Pretreatment of liver, lung, and ischial metastases before axitinib (arrows). (E–H) Two months after starting axitinib, liver, lung, and ischial metastases were evaluated; liver and lung metastases showed a reduction in size. The size of the metastatic lesion in the ilium was unchanged. (I, J) At 15 months after the initiation of treatment with axitinib, liver metastases had regressed. “I, J” indicates a newly developed liver metastasis. (K, L) At 15 months after starting axitinib, both lung and ischial metastases showed an increase in size.

**FIGURE 4:**
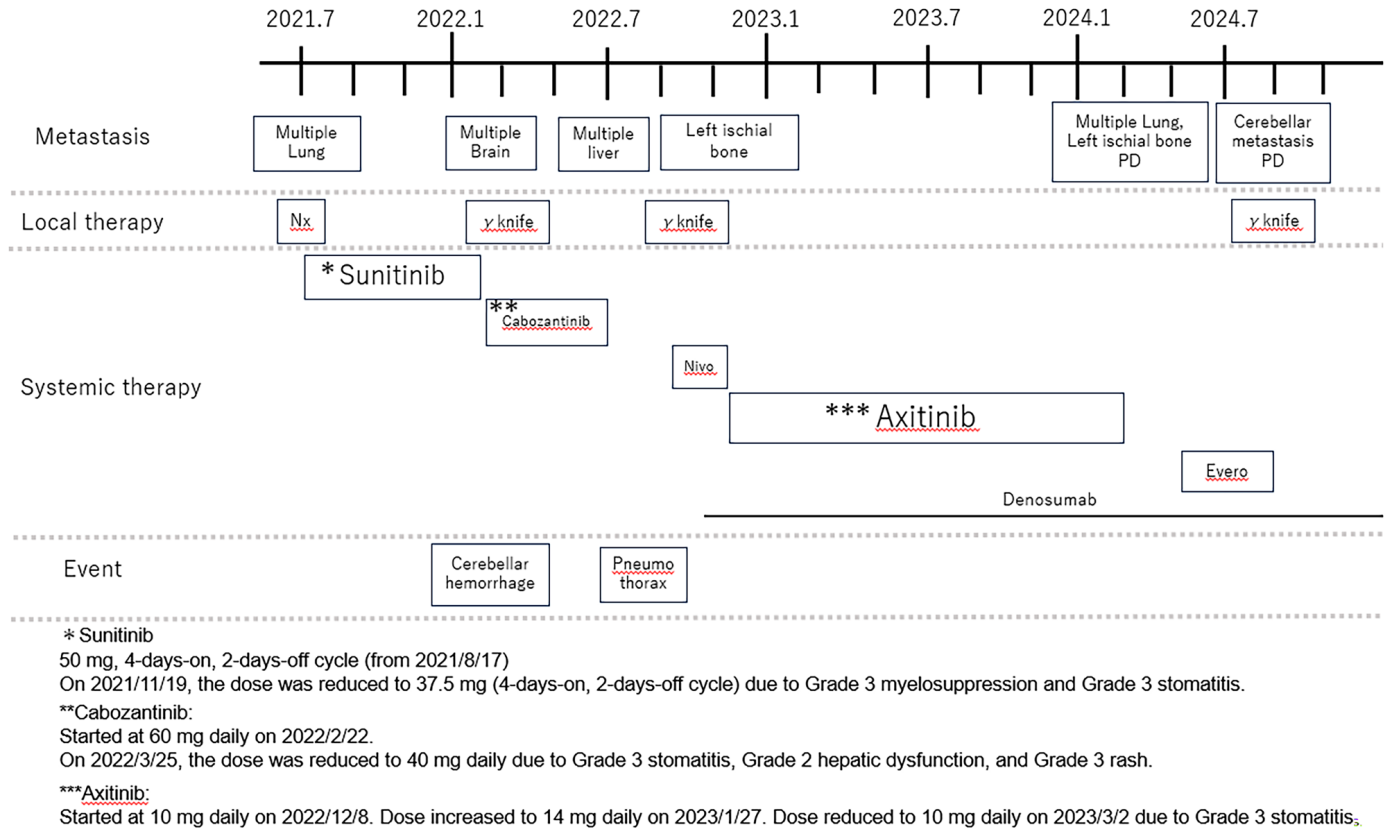
Timeline of the clinical course. Evero, everolimus; Nivo, nivolumab; PD, progressive disease.

Seven months later, second‐line cabozantinib (60 mg) was administered until the appearance of multiple liver metastases. Hence, administration of third‐line nivolumab (240 mg every 2 weeks) was initiated. Two months later, analysis revealed exacerbation of the liver, lung, and sciatic metastases (Figure 3A–D). Therefore, fourth‐line axitinib (10 mg/day) was initiated. Two months later, the liver and lung metastases had shrunk (Figure 3E–H). At 15 months after the initiation of treatment with axitinib, an increase in lung, liver, and sciatic metastases was observed (Figure 3I–L). Everolimus was administered as fifth‐line treatment for 5 months. Thereafter, the patient received best supportive care (Figure 4).

## 3 Discussion

PRCC is the most common subtype of nccRCC, accounting for approximately 15% of all renal cell carcinomas. The overall survival (OS) of patients with metastatic PRCC ranges from 12 to 22.5 months [2, 3].

Traditionally, PRCC has been classified into types 1 and 2 based on findings such as the degree of atypia, amount of cytoplasm, and pseudostratification of nuclei. Generally, Type 2 is linked to a poorer prognosis than Type 1. In the 2022 WHO Classification of Tumours (5th Edition), the classification of PRCC into types 1 and 2 was eliminated [1]. This patient was diagnosed in 2021 according to the 2016 WHO classification of kidney tumors.

At present, there is no established systemic treatment for advanced PRCC. The National Comprehensive Cancer Network guidelines (2021 version) recommended clinical trials or sunitinib for advanced nccRCC [4].

In the ASPEN and ESPEN trials involving patients with nccRCC, treatment with sunitinib resulted in median progression‐free survival (PFS) and OS of 6–8.3 and 16–32 months, respectively [5, 6]. In the National Comprehensive Cancer Network guidelines (2025 version), cabozantinib as first‐line treatment for PRCC did not show OS benefit compared with sunitinib (median PFS and OS were 9 and 20 months, respectively) [7, 8]. In this case, PFS was 6 and 7months with sunitinib and second‐line cabozantinib, respectively, consistent with the results of clinical trials.

In a retrospective study, nivolumab therapy for metastatic PRCC was associated with an objective response rate of 49% and a median PFS of 3.5 months [9]. The PFS in this case was 2 months with nivolumab, which was similar to that reported in previous cases.

Motzer et al., Tannir et al., and Yang et al. reported on the efficacy of axitinib for nccRCC, including papillary subtypes [10, 11, 12].

In our patient, fourth‐line axitinib led to a particularly long response.

Axitinib has been effective against metastatic PRCC in only a few cases, with three reports of successful use in the second‐line treatment for 8–16 months [1, 13, 14]. In these reports, the first‐line treatments were use of sunitinib (two cases) and temsirolimus (one case).

In the present case, axitinib as fourth‐line treatment after the prior use of two tyrosine kinase inhibitors resulted in a long‐term response of 15 months. Sunitinib selectively acts on vascular endothelial growth factor receptor 2 (VEGFR‐2), platelet‐derived growth factor receptor alpha, and KIT [15]. Cabozantinib selectively acts on VEGFR‐2, MET, and AXL receptor tyrosine kinases [16]. Axitinib selectively inhibits VEGFR‐1, VEGFR‐2, and VEGFR‐3 [17]. Bierer et al. and Ljungberg et al. suggested that VEGFR‐1, VEGFR‐2, and VEGFR‐3 are highly expressed in advanced PRCC (stage > 3) [18, 19]. In this case, the long‐term response to axitinib after the use of sunitinib and cabozantinib may be attributed to the high expression of VEGFR‐1 and VEGFR‐3 in the tumor. The histological type and genetic mutations of PRCC are heterogeneous. Moreover, the relationship between these mutations and the overexpression of VEGFR or drug efficacy remains unclear [13]. Thus, additional clinical reports combined with genetic testing are awaited.

## 4 Conclusion

Late‐sequence axitinib after the use of two types of tyrosine kinase inhibitors resulted in a long‐term response in the systemic treatment for metastatic PRCC. Axitinib is a useful option in the treatment sequence for advanced PRCC.

## Disclosure

The authors have nothing to report.

## Ethics Statement

The authors have nothing to report.

## Consent

The authors have nothing to report.

## Conflicts of Interest

The authors declare no conflicts of interest.

## Data Availability

The data that support the findings of this study are openly available in in Zenodo at https://doi.org/10.5281/zenodo.17581111.

## References

[1] World Health Organization (WHO) Classification of Tumors Editorial Board , WHO Classification of Tumors, 5th Edition, Volume 8 (International Agency for Research on Cancer, 2022).

[2] Y. Arai , Y. Kitamura , K. Miyai , et al., “Long‐Term Disease Control of Metastatic Type 2 Papillary Renal Cell Carcinoma Using Local Treatment and Molecular Targeted Therapy: A Case Report,” Molecular and Clinical Oncology 14 (2021): 71. 33732457 10.3892/mco.2021.2233 PMC7907800

[3] K. Ito , S. Mikami , K. Tatsugami , et al., “Clinical Outcomes in Patients With Metastatic Papillary Renal‐Cell Carcinoma: A Multi‐Institutional Study in Japan,” Clinical Genitourinary Cancer 16 (2018): e1201–e1214. 30224330 10.1016/j.clgc.2018.07.028

[4] National Comprehensive Cancer Network (NCCN) , “NCCN Guidelines Version 1: Kidney Cancer” (2021).

[5] A. J. Armstrong , S. Halabi , T. Eisen , et al., “Everolimus Versus Sunitinib for Patients With Metastatic Non‐Clear‐Cell Renal Cell Carcinoma (ASPEN): A Multicenter, Open‐Label, Randomized Phase 2 Trial,” Lancet Oncology 17 (2016): 378–388. 26794930 10.1016/S1470-2045(15)00515-X PMC6863151

[6] N. M. Tannir , E. Jonasch , L. Albiges , et al., “Everolimus Versus Sunitinib Prospective Evaluation in Metastatic Non‐Clear Cell Renal Cell Carcinoma (ESPN): A Randomized Multicenter Phase 2 Trial,” European Urology 69 (2016): 866–874. 26626617 10.1016/j.eururo.2015.10.049 PMC4879109

[7] National Comprehensive Cancer Network (NCCN) , “Guidelines Version 3: Kidney Cancer” (2025).

[8] S. K. Pal , C. Tangen , I. M. Thompson , et al., “A Comparison of Sunitinib With Cabozantinib, Crizotinib, and Savolitinib for Treatment of Advanced Papillary Renal Cell Carcinoma: A Randomized, Open‐Label, Phase 2 Trial,” Lancet 397 (2021): 695–703. 33592176 10.1016/S0140-6736(21)00152-5 PMC8687736

[9] V. S. Koshkin , P. C. Barata , T. Zhang , et al., “Clinical Activity of Nivolumab in Patients With Non‐Clear Cell Renal Cell Carcinoma,” Journal for Immunotherapy of Cancer 6 (2018): 9. 29378660 10.1186/s40425-018-0319-9 PMC5789686

[10] R. J. Motzer , B. Escudier , P. Tomczak , et al., “Axitinib Versus Sorafenib as Second‐Line Treatment for Advanced Renal Cell Carcinoma: Overall Survival Analysis and Updated Results From a Randomized Phase 3 Trial,” Lancet Oncology 14 (2013): 552–562. 23598172 10.1016/S1470-2045(13)70093-7

[11] N. M. Tannir , S. P. Pal , and M. B. Atkins , “Second‐Line Treatment Landscape for Renal Cell Carcinoma: A Comprehensive Review,” Oncologist 23 (2018): 540–555. 29487224 10.1634/theoncologist.2017-0534 PMC5947457

[12] Y. Yang , S. P. Psutka , A. B. Parikh , et al., “Combining Immune Checkpoint Inhibition Plus Tyrosine Kinase Inhibition as First and Subsequent Treatments for Metastatic Renal Cell Carcinoma,” Cancer Medicine 11 (2022): 3106–3114. 35304832 10.1002/cam4.4679 PMC9385597

[13] G. Ishii , T. Hatano , K. Endo , et al., “A Case of Papillary Renal Cell Carcinoma Type 2 Resistant to Sunitinib Responded to Second Line Therapy With Axitinib” [in Japanese], Nihon Hinyokika Gakkai Zasshi 105 (2014): 129–133. 25158555 10.5980/jpnjurol.105.129

[14] J. Y. Kim , H. O. Jeong , D. S. Heo , et al., “Treatment Strategy for Papillary Renal Cell Carcinoma Type 2: A Case Series of Seven Patients Treated Based on Next Generation Sequencing Data,” Annals of Translational Medicine 8 (2020): 1389. 33313134 10.21037/atm-20-3466 PMC7723617

[15] S. Faivre , G. Demetri , W. Sargent , and E. Raymond , “Molecular Basis for Sunitinib Efficacy and Future Clinical Development,” Nature Reviews. Drug Discovery 6 (2007): 734–745. 17690708 10.1038/nrd2380

[16] F. M. Yakes , J. Chen , J. Tan , et al., “Cabozantinib (XL184), a Novel MET and VEGFR2 Inhibitor, Simultaneously Suppresses Metastasis, Angiogenesis, and Tumor Growth,” Molecular Cancer Therapeutics 10 (2011): 2298–2308. 21926191 10.1158/1535-7163.MCT-11-0264

[17] B. I. Rini , B. Escudier , P. Tomczak , et al., “Comparative Effectiveness of Axitinib Versus Sorafenib in Advanced Renal Cell Carcinoma (AXIS): A Randomized Phase 3 Trial,” Lancet 378 (2011): 1931–1939. 22056247 10.1016/S0140-6736(11)61613-9

[18] S. Bierer , E. Herrmann , T. Kopke , et al., “Lymphangiogenesis in Kidney Cancer: Expression of VEGF‐C, VEGF‐D and VEGFR‐3 in Clear Cell and Papillary Renal Cell Carcinoma,” Oncology Reports 20 (2008): 721–725. 18813809

[19] B. J. Ljungberg , J. Jacobsen , S. H. Rudolfsson , et al., “Different Vascular Endothelial Growth Factor (VEGF), VEGF‐Receptor 1 and ‐2mRNA Expression Profiles Between Clear Cell and Papillary Renal Cell Carcinoma,” BJU International 98 (2006): 661–667. 16925769 10.1111/j.1464-410X.2006.06387.x

